# Using caching and optimization techniques to improve performance of the Ensembl website

**DOI:** 10.1186/1471-2105-11-239

**Published:** 2010-05-11

**Authors:** Anne Parker, Eugene Bragin, Simon Brent, Bethan Pritchard, James A Smith, Stephen Trevanion

**Affiliations:** 1 Wellcome Trust Sanger Institute, Wellcome Trust Genome Campus, Hinxton, Cambs CB10 1SA, UK

## Abstract

**Background:**

The Ensembl web site has provided access to genomic information for almost 10 years. During this time the amount of data available through Ensembl has grown dramatically. At the same time, the World Wide Web itself has become a dramatically more important component of the scientific workflow and the way that scientists share and access data and scientific information.

Since 2000, the Ensembl web interface has had three major updates and numerous smaller updates. These have largely been in response to expanding data types and valuable representations of existing data types. In 2007 it was realised that a radical new approach would be required in order to serve the project's future requirements, and development therefore focused on identifying suitable web technologies for implementation in the 2008 site redesign.

**Results:**

By comparing the Ensembl website to well-known "Web 2.0" sites, we were able to identify two main areas in which cutting-edge technologies could be advantageously deployed: server efficiency and interface latency. We then evaluated the performance of the existing site using browser-based tools and Apache benchmarking, and selected appropriate technologies to overcome any issues found. Solutions included optimization of the Apache web server, introduction of caching technologies and widespread implementation of AJAX code. These improvements were successfully deployed on the Ensembl website in late 2008 and early 2009.

**Conclusions:**

Web 2.0 technologies provide a flexible and efficient way to access the terabytes of data now available from Ensembl, enhancing the user experience through improved website responsiveness and a rich, interactive interface.

## Background

### What is Web 2.0?

Since its definition in 2004 [1], Web 2.0 has been a much-touted buzzword. Originally intended only to mark the "resurrection" of the web in the wake of the dot-com meltdown, it is now seen as a distinctive approach to web development, typified by the following factors:

• Large online data sources

• User interaction and collaboration

• Rich web-based interfaces

In this review we will be focusing on the first of these aspects: improvements to the speed and efficiency of serving large datasets.

### Large data sources

**Figure 1:**
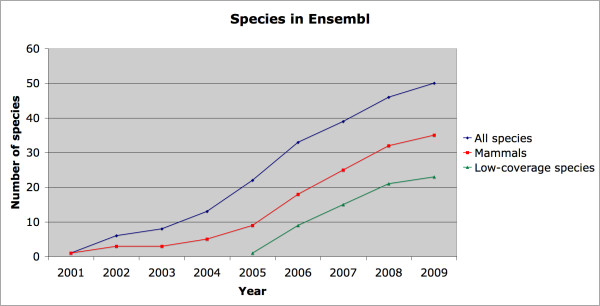
**Increase in species displayed in Ensembl**. The number of species included in the Ensembl release has risen rapidly, from only one (human) in the original release to fifty in 2009. Each new species not only adds arithmetically to the amount of data in the main species database, but also geometrically to any cross-species analyses such as the Ensembl Compara databases. Lower-coverage genomes generally produce smaller databases but are more numerous than the high-coverage genomes. In addition, existing species may gain new data types, for example variation data, further increasing the demand on storage space.

Typical Web 2.0 data sources are the map and satellite images underlying Google Maps, Amazon's book inventory, or Wikipedia's hundreds of thousands of articles. Likewise Ensembl contains information -- DNA sequence, gene annotations, variation and functional genomics data -- on a scale barely imaginable ten years ago; recent releases have comprised over half a terabyte of MySQL files. The number of species, and thus the amount of data being served, continues to rise year on year (Figure 1), whilst physical resources (including both hardware and the electricity needed to run it) struggle to keep up, making it imperative for Ensembl to access databases and serve pages as efficiently as possible [2].

## Methods

We researched the technologies being used by Web 2.0 sites to enhance their performance, and chose those that were most compatible with our existing server setup. We also selected a number of free development tools that could be used to assess website responsiveness.

### Caching technologies

One of the principle problems when running a popular website is the time taken to fetch data from filesystems and databases and present the resultant pages to the many users who are simultaneously accessing the site. Matters can be improved up to a point by adding more hardware, but then there is the issue not just of cost but also of co-ordinating data across multiple machines so that the user experience is seamless. Prior to the site redesign, Ensembl used a single filesystem shared across multiple webservers using the General Parallel File System (GPFS) created by IBM. This allowed the web files to be stored in only one place whilst many separate CPUs ran Apache webserver processes to return the data to hundreds of simultaneous users.

However as the site grew ever larger and more heavily used, GPFS was found to be unstable when dealing with large numbers of concurrent users. This is a problem that is intrinsic to shared filesystems, and so an alternative solution was needed. Memcached [3] is a high-performance, distributed memory object caching system. It was created for LiveJournal.com as a way of dealing with very high website usage (>20 million dynamic page views per day), and allows web files to be stored in a shared memory space and re-served to site visitors without needing to go back to the file system. A cluster of servers has access to this shared memory space, which thus acts like a shared file system but without either data access latency or instability under high load. Popular files will tend to stay in memory almost permanently, with others being cycled in and out of memory as they are needed. For efficiency of memory management, memcached enables each file to be tagged with an identifier, so that whole categories of data can be flushed from memory when the information stored on the hard drives is updated.

File access time is only part of the problem, however. The Ensembl web code loads large quantities of Perl into Apache, via mod_perl, in order to access its several dozen databases in an efficient manner, with the result that each Apache process occupies around 300 Mb of RAM. Even a powerful server with 16 Gb of RAM cannot run more than fifty such processes at a time, and with each web page being made up of an average of 10-15 files, only a few complete pages can be served each second. In addition, large slow requests tend to be queued behind faster ones, resulting in longer and longer response times as the number of concurrent users increases.

These large Apache processes are necessary for accessing data from the genomic database to build dynamic displays, but they are overkill when it comes to serving small static files such as template images and text-based files such as HTML and CSS. We therefore implemented nginx [4], a free, open-source, high-performance HTTP server and reverse proxy, which integrates well with memcached. Nginx takes over the role of serving the static files, so that only the requests that need the full might of an Apache process get passed back to the main servers.

### Client-side vs server-side testing

Both client-side and server-side tools were used in the analysis. Server-side tests have the advantage that they apply specifically to the server hardware in use and should therefore be consistent for all users, but they cannot measure the interactions between server and client, which is a major element of any web site's performance. Client-side tests, on the other hand, will vary enormously in their results, depending upon both the setup of the client machine and its browser and the speed of the network connection to the server. Consequently the relative improvement between optimised and non-optimised sites is more important than the actual figures involved.

In all cases, the client-side tests were made using the Firefox web browser running under Windows (a typical usage scenario) from machines within the Sanger Institute; absolute response times are therefore likely to be a good deal faster than external usage but, as explained above, this does not invalidate the relative improvements produced by caching and optimisation technologies; on the contrary, users on slower connections may see substantially greater improvements.

### Tools used in analysis and testing

#### Firebug

Firebug [5] is a plugin for the Firefox web browser, which allows detailed analysis of HTTP requests, both in terms of the content returned (useful for debugging) and the time taken to return each page component. It was this latter feature that was of particular use in our development process.

We use Firebug extensively throughout our development process, mainly to debug AJAX requests. In the case of optimisation testing, we analysed a number of different pages and obtained broadly similar results; for the sake of simplicity and clarity, only one sample page analysis is included in this paper.

#### YSlow

Whilst improvements in the underlying code are essential, there are some simple steps that can be taken to increase website efficiency significantly with relatively little work. Many of these take advantage of the changes in Internet technology in the last ten to fifteen years; the optimisations that worked well back in the days of dial-up can actually be counter-productive in an era of widespread broadband usage.

YSlow [6] is an addition to the Firebug plugin which rates a web page's efficiency. The underlying concepts were developed by Yahoo!'s Exceptional Performance team, who identified 34 rules that affect web page performance. YSlow's web page analysis is based on the 22 of these 34 rules that are testable.

Only a single test before optimisation, and another afterwards, was required, since YSlow focuses on general characteristics of the site rather than individual pages.

#### Apache benchmarking

The Apache webserver comes with a benchmarking program, *ab *[7], which can be used to analyze server performance and produce statistics on how many requests per second can be served. We ran two sets of tests, one for memcached and one for nginx. For memcached, which is designed to improve performance of dynamic content, we used the standard "Region in Detail" view from Ensembl Human; for nginx, we used the Ensembl home page, which contains a number of static files, including several images. We ran each battery of tests first with the given technology turned off, and then with it turned on. For speed we chose to configure the benchmark program to make only 100 requests at a time; adding more requests increases the accuracy slightly, but causes the non-cached tests to take a long time. The tests were run repeatedly using different parameters for the number of concurrent users (1, 10, 20, 30 and so on up to 100), and the relevant figures from the benchmark output were recorded in a spreadsheet.

## Results and Discussion

### Apache optimization

**Figure 2:**
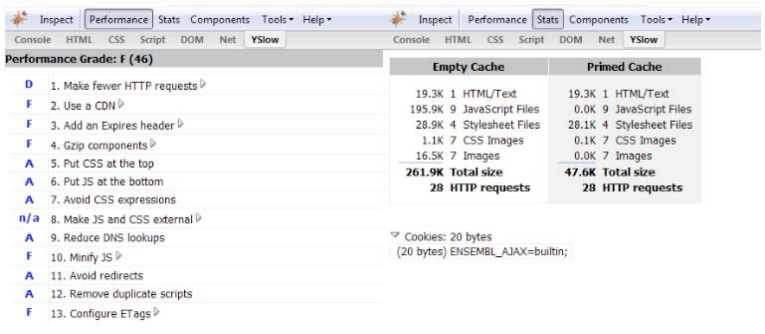
**YSlow analysis of Ensembl home page before changes**. Combined screenshots of YSlow in the Firebug window, showing the 'Performance' and 'Stats' displays respectively. On the left are the factors assessed by YSlow, with a score next to each; the overall score (F) and percentage (46%) are shown at the top of the column. On the right is a table of files downloaded for the page, with file sizes and HTTP request totals. Note how all 28 requests were repeated, even though browser caching of JavaScript and images meant that fewer kilobytes of data were actually downloaded.

An initial analysis of the Ensembl home page using YSlow gave the following results (Figure 2), scoring the Ensembl site as 'F', the lowest possible. Our sample page returned 28 separate files totalling 261.9 KB of data on the first visit and 47.6 Kb after browser caching. Also, despite browser caching, 28 HTTP requests were made on every page visit, representing a substantial load on the server. The five main areas we addressed, as highlighted by the YSlow grading, were:

1. Number of HTTP requests

2. Changes to 'Expires' headers

3. Gzipping of page components

4. Minifying JavaScript

5. Configuring ETags

YSlow also suggested using a content delivery network (CDN), which deploys content via a geographically dispersed network of servers. This was not deemed appropriate for Ensembl, as the cost of such a service could not be justified in terms of performance (we have however begun deploying international mirror sites as a way of overcoming network latency).

HTTP requests were reduced in the first place by deploying Perl code that, on server startup, merges the separate CSS stylesheets into a single .css file and likewise the JavaScript modules into a single .js file. This immediately eliminated 11 of the 28 HTTP requests. The Perl script also "minifies" both CSS and JavaScript by eliminating all unnecessary whitespace, thus addressing point 4.

The 'Expires' header in a file served via HTTP tells the browser when to check to see if a file has been updated. Most of the Ensembl template files do not change from release to release, so by adding an 'Expires' header of one month we can ensure that visitors' browsers do not make unnecessary requests for files that are unchanged. This further reduces the number of HTTP requests on subsequent page visits. The minified CSS and JavaScript files are renamed each time they are changed, so that the browser treats them as new files and fetches them again; however this normally happens only once per release, so has minimal impact on performance. Gzipping was implemented by enabling the mod_deflate compression module within Apache, so that all files are automatically sent in a compressed form if the browser indicates that it can handle such files.

Etags are another method of telling the browser whether the file has been changed, by attaching a unique string to the header. Normally this feature is turned on automatically, with the result that the browser always makes an HTTP request to confirm whether the file has changed or not before downloading it. This made sense in the days of slow dialup web connections, but with the spread of broadband, the brief time taken to download a typical web page component no longer justifies the server overhead.

**Figure 3:**
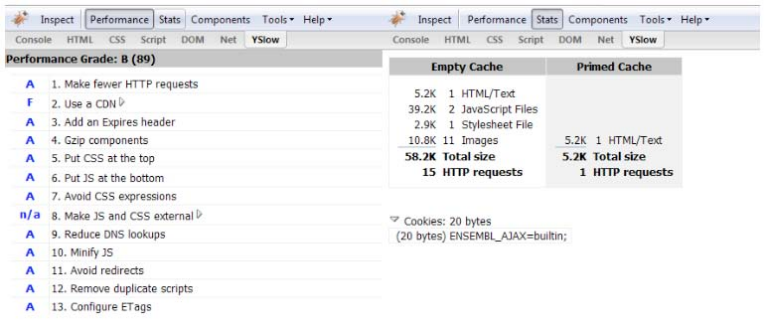
**YSlow analysis of Ensembl home page after changes**. Combined screenshots of YSlow in the Firebug window, showing the 'Performance' and 'Stats' displays respectively. On the left are the factors assessed by YSlow, with a score next to each; the overall score (B) and percentage (89%) are shown at the top of the column. On the right is a table of files downloaded for the page, with file sizes and HTTP request totals. In the optimized page, cached files no longer triggered HTTP requests, resulting in a single request for the HTML file itself.

By turning off Etags in the Apache configuration, HTTP requests can be further reduced with no discernable impact on the user. After optimization, the site was scored as 'B' (Figure 3), a substantial improvement. The same page returned only 15 files and 58.2 Kb of data on the first visit, and only one file of 5.2 Kb on a subsequent visit.

### Caching and proxying technologies

Both memcached and nginx produced marked improvement in the performance of the server.

**Figure 4:**
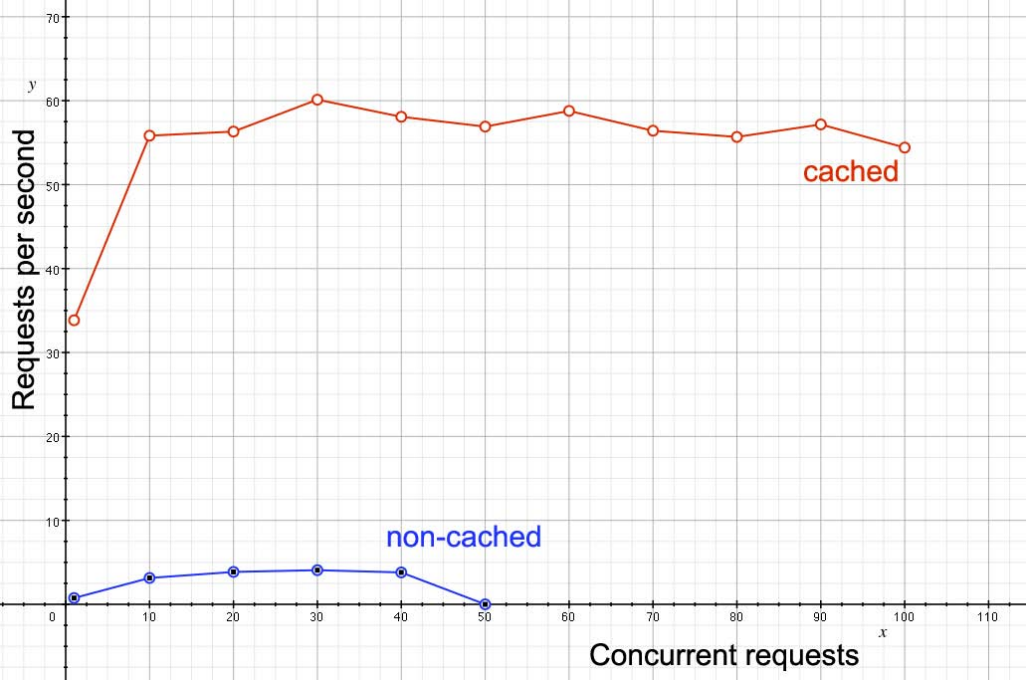
**Benefits of caching dynamically generated content**. A plot of returned requests per minute against number of concurrent users, showing the marked performance difference of the Apache server alone (lower blue line) and the memcached-enable server (upper red line).

The following Apache benchmark graph demonstrates the benefits of using memcached. In Figure 4, server responses returned per second is plotted against the number of concurrent users accessing the site, for dynamic pages that access the databases to build displays of genomic data. The lower blue line shows how the site performs when no caching is done; at no point does responsiveness exceed three requests per second, and once fifty concurrent users are reached, the site grinds to a halt. By contrast, the memcached-enabled site not only returns far more requests per second, but performance is not affected by the number of concurrent users.

**Figure 5:**
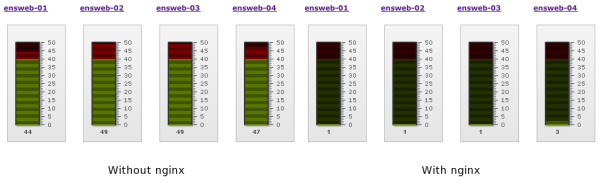
**Apache server load with and without nginx**. On the left are four Ensembl servers running Apache without the benefit of nginx. All four are in the red "danger" zone of excessive load, with 44-49 out of 50 available processes in use. On the right are the same four servers with nginx; all are now being very lightly used, with only 1-3 processes out of the 50 being required.

The following screenshots of our server monitoring software (Figure 5) show the loads on four sample server nodes with and without nginx. In both cases the scale represents the number of Apache processes, with the lower green area being a safe load and the upper red area being excessive load. As can be seen, without nginx all the servers are operating at maximum or near-maximum capacity, whereas with nginx the servers are idling, with only 1-3 processes in use. This allows us to cope with peaks of usage even on relatively modest amounts of hardware.

**Figure 6:**
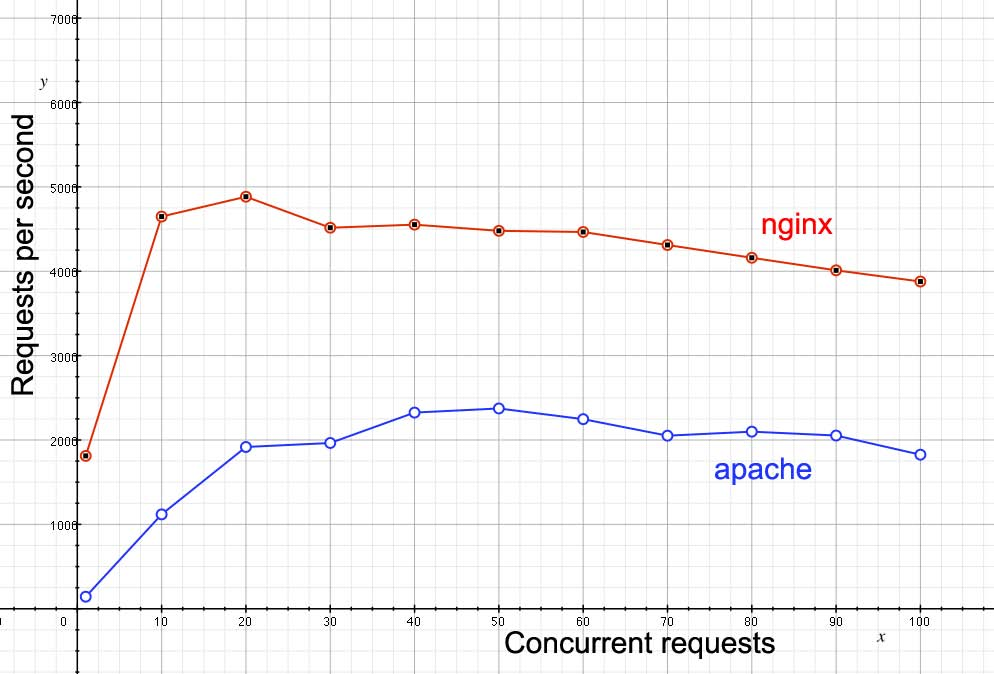
**Comparison of responsiveness with and without nginx**. A plot of returned requests per minute against number of concurrent users for static files, showing the marked performance difference of the Apache server alone (lower blue line) and the nginx-enable server (upper red line).

A graph of response times (Figure 6) tells a similar story to that of memcached, but for static rather than dynamic content. With nginx enabled, the server's power can be focused on returning data-heavy content, and so the static files are typically returned to the user twice as fast as with Apache using memcached alone.

The combination of memcached and nginx has had an enormous impact on the Ensembl website, improving the user experience by serving pages far faster than was previously possible and without consuming additional resources.

### AJAX

The traditional method of creating and serving dynamic web pages is to fetch all the data from the database, create the HTML and images, and only then send the web page back to the user's browser. For small pages this is no problem, but when complex database queries are involved, there is often a substantial delay between the user clicking on a link and the page starting to appear in the browser. This is frustrating to the user [8], who may be left staring at a blank screen for several seconds, or even a minute or more in the case of very large and complex requests.

**Figure 7:**
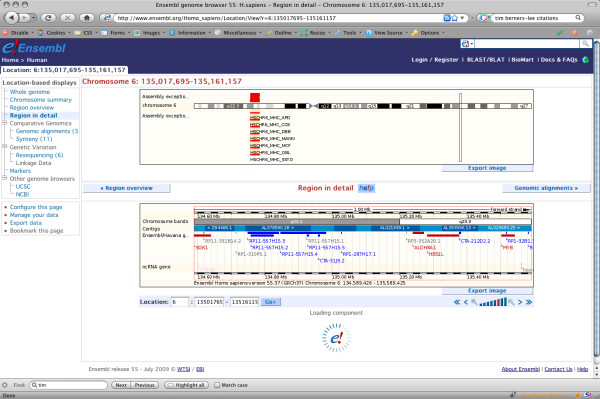
**AJAX loading of data-heavy content**. When a data-heavy Ensembl page such as 'Region in Detail" is requested, the server immediately returns the page template with its navigation links, plus cached images such as small-scale overviews. The main image, however, is generated in the background, and a placeholder graphic (the e! logo within an animated spinner) is shown instead. After a few seconds the placeholder will disappear and the full image is loaded onto the page.

AJAX (Asynchronous Javascript And XML) [9] offers an alternative method of returning and refreshing web pages so that they appear to the user to be faster and more responsive. Rather than waiting for the whole page to be built before returning any data, AJAX-enabled pages can return the data-light sections of the page such as the header and navigation immediately, then insert the images, tables and other "data heavy" page components as they become available (Figure 7). The Ensembl website originally used the Scriptaculous and Prototype AJAX libraries [10], but has since moved over to using jQuery [11], as the codebase for the functionality required by Ensembl is smaller in the latter.

[Note that despite the standard acronym for this technique, Ensembl actually uses XHTML rather than XML, to avoid the overhead of parsing XML into a browser compatible format.]

**Figure 8:**
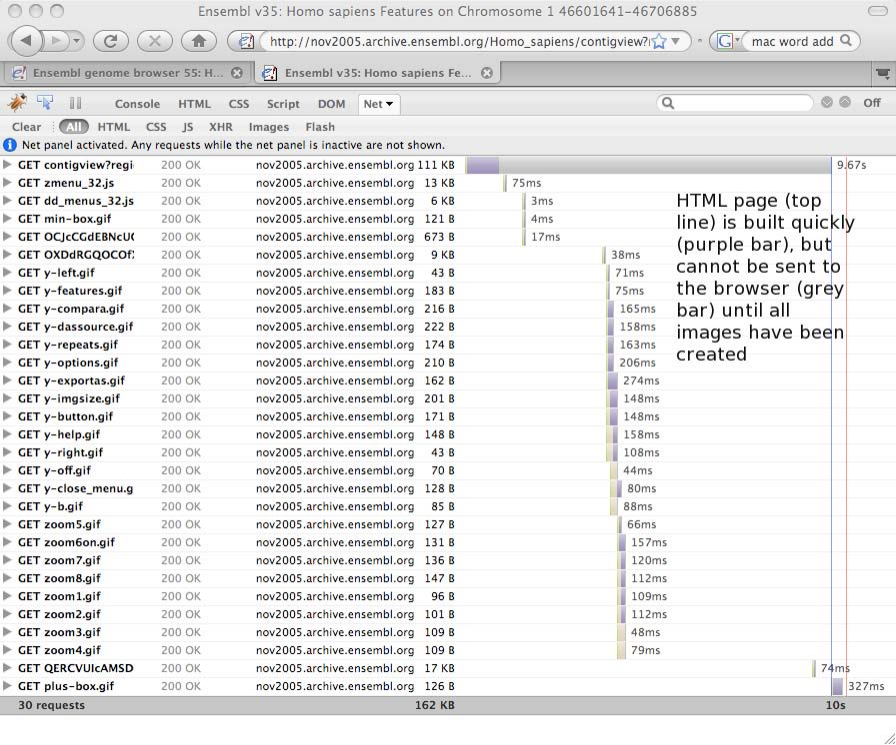
**Load times for a non-AJAX page**. A screenshot of the Firebug analysis of download times for a typical non-AJAX page. No content is sent to the browser until all the images have been generated, as indicated by the red 'load' line at the end of the graph.

**Figure 9:**
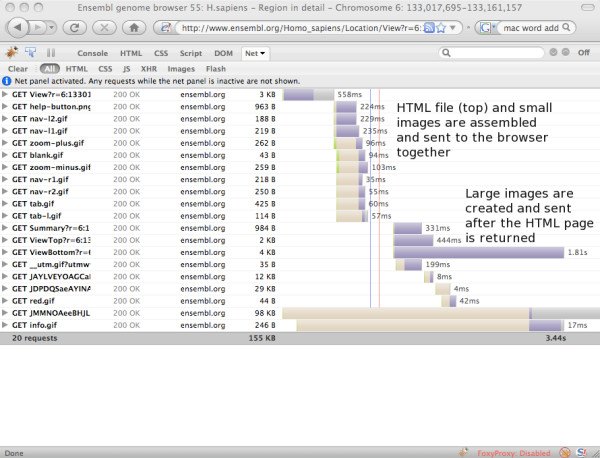
**Load times for an AJAX-enabled page**. A screenshot of the Firebug analysis of download times for a typical AJAX-enabled page. Static files are sent to the browser first, as indicated by the red 'load' line which falls immediately after the time bar for the last .gif file used by the sequence navigation, then dynamic images are sent as they become available.

The screenshots below show the Firebug diagnostic tools display for download times, first for an archive site that lacks AJAX (Figure 8.), then for an equivalent page on the current (55) release (Figure 9). Note that the overall amount of data being downloaded is very similar; however the page loads substantially faster in the new version, due in large part to the other improvements already described. Where AJAX contributes is not so much in absolute load times as in perceived load times. Whereas the non AJAX page is not sent to the user until every file is available, some ten seconds after the page was requested, the AJAX page begins returning content after only one second, as indicated by the vertical red line that marks the point where a response is sent back to the requestor. Feedback to the user is thus much more immediate, leading to an improved browsing experience.

## Conclusions

The size and complexity of genomic datasets now being produced demand a more flexible and imaginative approach to web application development within science, and the use of technologies that have hitherto lain outside the purview of the scientific community. We have shown how the adoption of these technologies can be of enormous benefit to the users of online scientific resources, allowing vast datasets to be served to the user with minimal latency.

## Authors' contributions

EB installed and tested memcached and nginx; JS ran the YSlow tests and implemented minification. JS also wrote the original AJAX code, which was subsequently ported to jQuery by SB, and all authors were involved in updating the web code to use this functionality and deploying the new site. AP wrote this paper; images and figures were created by AP, EB and JS. All authors read and approved the final manuscript.
